# Buccal Fat Pad as a Potential Source of Stem Cells for Bone Regeneration: A Literature Review

**DOI:** 10.1155/2017/8354640

**Published:** 2017-07-05

**Authors:** Nasim Salehi-Nik, Maryam Rezai Rad, Lida Kheiri, Pantea Nazeman, Nasser Nadjmi, Arash Khojasteh

**Affiliations:** ^1^Department of Tissue Engineering, School of Advanced Technologies in Medicine, Shahid Beheshti University of Medical Sciences, Tehran, Iran; ^2^Dental Research Center, Research Institute of Dental Sciences, School of Dentistry, Shahid Beheshti University of Medical Sciences, Tehran, Iran; ^3^School of Dentistry, Shahid Beheshti University of Medical Sciences, Tehran, Iran; ^4^University of Antwerp, The Team for Cleft and Craniofacial Anomalies, Oral and Maxillofacial Surgery, University of Antwerp, Antwerp, Belgium; ^5^Medical Nano-Technology & Tissue Engineering Research Center, Shahid Beheshti University of Medical Sciences, Tehran, Iran; ^6^Faculty of Medicine, University of Antwerp, Antwerp, Belgium

## Abstract

Adipose tissues hold great promise in bone tissue engineering since they are available in large quantities as a waste material. The buccal fat pad (BFP) is a specialized adipose tissue that is easy to harvest and contains a rich blood supply, and its harvesting causes low complications for patients. This review focuses on the characteristics and osteogenic capability of stem cells derived from BFP as a valuable cell source for bone tissue engineering. An electronic search was performed on all in vitro and in vivo studies that used stem cells from BFP for the purpose of bone tissue engineering from 2010 until 2016. This review was organized according to the PRISMA statement. Adipose-derived stem cells derived from BFP (BFPSCs) were compared with adipose tissues from other parts of the body (AdSCs). Moreover, the osteogenic capability of dedifferentiated fat cells (DFAT) derived from BFP (BFP-DFAT) has been reported in comparison with BFPSCs. BFP is an easily accessible source of stem cells that can be obtained via the oral cavity without injury to the external body surface. Comparing BFPSCs with AdSCs indicated similar cell yield, morphology, and multilineage differentiation. However, BFPSCs proliferate faster and are more prone to producing colonies than AdSCs.

## 1. Introduction

Mesenchymal stem cells (MSCs) derived from bone marrow aspirates have been frequently used as a cell source in bone tissue engineering [1]. However, several problems are associated with the clinical application of bone marrow stem cells (BMSCs) [1]. The harvesting procedure is associated with pain and discomfort for patients, and their differentiation capability is dependent on the donor age [2].

Adipose tissues have been introduced as a promising source of MSCs that can be obtained with minimal discomfort for patients, since subcutaneous adipose tissues are usually discarded after aesthetic surgical procedures. In addition, several studies have shown that the cell yield from adipose tissues is 100 to 500 times greater than that from bone marrow aspirates [3–5]. Therefore, minimally invasive procedures can be used to obtain a high number of MSCs with similar multilineage capabilities [6–8]. However, not all patients undergo liposuction, and fat distribution is dependent on body weight.

Recently, Farre-Guasch et al. isolated adipose-derived stem cells (AdSCs) from a mass of fatty tissue in the oral cavity called Bichat's fat pad or the buccal fat pad (BFP). These cells have a similar phenotype to AdSCs from abdominal subcutaneous adipose tissue [9]. Under appropriate conditions, AdSCs derived from BFP (BFPSCs) have been shown to differentiate into chondrocytes, osteoblasts, or adipocytes in vitro [9]. Moreover, Shiraishi et al. reported that BFPSCs can form engineered bone in the back subcutaneous pockets of nude mice [10]. Khojasteh and Sadeghi recently used BFPSCs in conjunction with iliac bone block grafts and showed an increase in the amount of new bone formation and a decrease in secondary bone resorption in extensively atrophic jaws [11].

In addition to BFPSCs, dedifferentiated fat cells (DFAT) derived from BFP (BFP-DFAT) can be produced from mature adipocytes by a convenient method called ceiling culture technique. These cells possess high potential for regeneration of the bone and periodontal tissues [10, 12]. Therefore, BFP could be considered as a potential cell source for bone engineering in oral and craniofacial areas since it is easy to harvest and provides a proper quantity of tissue for cell isolation. The present study reviews research on the characteristics and osteogenic capability of stem cells derived from BFP as a promising cell source for bone tissue engineering in the oral and craniofacial regions.

## 2. Materials and Methods

This systematic review has been organized according to the preferred reporting items for systematic reviews and meta-analysis (PRISMA) statement.

### 2.1. Eligibility Criteria

This review included all in vitro and in vivo studies that used BFPSCs and BFP-DFAT cells from human or animal sources for bone regeneration. Abstracts, reviews, letters, and theses were excluded. Studies were excluded if they used the BFP flap or mass (i.e., without cells) and if they did not focus on bone formation or differentiation towards the osteoblast lineage.

### 2.2. Search Strategy and Study Selection

The PubMed/MEDLINE, EMBASE, Web of Science, and Cochrane electronic databases were searched for relevant studies published between January 2010 and November 2016. No limitation in language was applied in the search process. The following search terms were used, in which “mh” represents the MeSH terms and “tiab” represents the title or abstract: (“buccal fat pad” (mh) or “buccal fat pad” (tiab) or “BFP” (mh) or “BFP” (tiab)) and (“cell” (tiab) or “stem cell” (tiab) or “tissue engineering” (tiab) or “adipose tissue stem cell” (tiab)). Additionally, a manual search was also performed in the following journals in the given time periods: Stem Cells, Stem Cell Research, Journal of Stem Cells, and Regenerative Medicine.

Initial screening of titles and abstracts was carried out based on the inclusion and exclusion criteria, and full texts of all eligible studies were obtained. Two independent reviewers extracted and processed data for analysis according to the predefined eligibility criteria. In case of any disagreement, agreement was obtained following a discussion with the third reviewer. The fourth and fifth reviewers contributed to discussion section.

### 2.3. Data Items

The results and data were extracted from the full text of the articles. The studies were then classified and summarized as in vitro and in vivo studies. In in vitro studies, the following outcomes were assessed: alkaline phosphate (ALP) activity, alizarin red staining, osteocalcin (OCN) content, and gene expression using reverse transcription polymerase chain reaction (RT-PCR). The results of histological evaluation and radiographic evaluation were also assessed for in vivo studies. Since the focus of the present review is the BFP stem cells derived from both human and animal origins, the sources of cells were also identified (Tables 1 and 2).

## 3. Results

**Figure 1:**
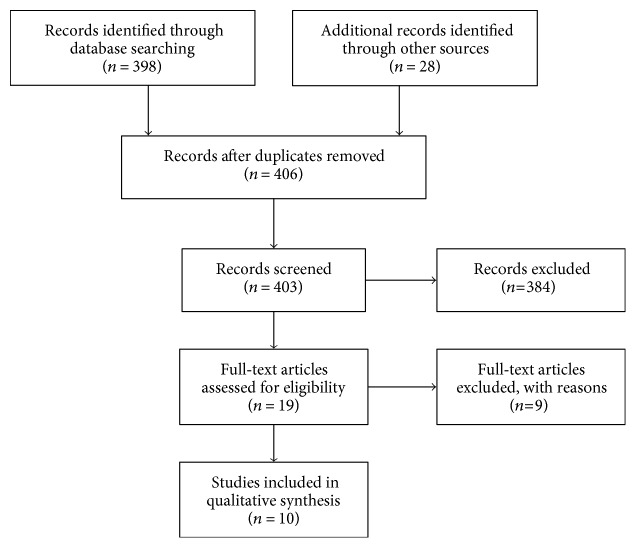
Search strategy flowchart.

As illustrated in the PRISMA flow diagram in Figure 1, the initial search retrieved a total of 406 studies. Following the initial screening of titles and abstracts, 16 studies were selected for final screening of the full texts. A total of 10 articles met the inclusion criteria and were included in the analysis (Figure 1). Of these 10 studies, seven were conducted in vitro [9, 12–17], and the other three were conducted both in vitro and in vivo [10, 11, 18].

All in vitro studies except for two compared BFPSCs with other cell sources, including AdSCs, BMSCs, unrestricted somatic stem cells (USSCs), and subcutaneous adipose stem cells (SC-AdSCs) [9, 12–14, 17]. All studies derived stem cells from human volunteers except for a study by Niada et al., where BFPSCs were derived from swine and compared to SC-AdSCs [13]. Three of the 10 studies focused on BFP-DFAT cells [14–16]. One experiment focused on the size of DFAT cells and compared small cells (<40 *μ*m) with large cells (40–100 *μ*m) based on the characteristics for MSCs [16].

Three studies reported in vivo results in addition to in vitro assessment [10, 11, 18]. Two studies reported bone formation results after the application of BFPSCs in animal models [10, 11], and one study performed human bone regeneration. Shiraishi et al. used recombinant human bone morphogenetic protein 2 (rhBMP2) with cells [10], and Nagasaki et al. combined low-intensity pulsed ultrasound and nanohydroxyapatite scaffolds for transplanting BFPSCs into the calvarial defects of mice [18]. Khojasteh and Sadeghi loaded BFPSCs on an allograft and implanted that in combination with autogenous iliac bone in severely atrophic jaws [11].

Data extracted on the characteristics of BFPSCs and SC-AdSCs are compared in Table 3. Tissue volume, number of cells, collagen deposition, ALP activity, and glycosaminoglycan (GAG) content have been shown to be greater in SC-AdSCs, but cell proliferation, morphology, size, adipogenic differentiation, and expression of MSCs markers are similar. In addition, unlike SC-AdSCs, BFPSCs are capable of producing colony-forming units [9, 12, 13].

## 4. Discussion

Adipose tissue contains two different fractions: (1) stromal vascular fraction (SVF), which includes MSCs (preadipocytes), fibroblasts, and erythrocytes and (2) mature adipocytes [9]. AdSCs isolated from the SVF were considered to be the key MSCs within this tissue and can be induced towards adipocytes, osteocytes, chondrocytes, myocytes, and neurons [19–21]. Since the SVF has a complex structure and cellular composition, AdSCs derived from SVF, particularly in early passages, are heterogeneous populations composed of cells with various characteristics and behaviors [9, 22–27].

Mature adipocytes are another abundant type of cells in fat tissue, and they have also shown dynamic plasticity to be converted into DFAT cells by a ceiling culture technique [28–30]. Unlike terminally differentiated adipocytes, DFAT cells have significant and steady proliferation capability [30–33]. In contrast to AdSCs, DFAT cells have been shown to have a more homogeneous cell population [15, 16, 30]. In addition, a greater number of DFAT cells can be produced from a given amount of fat tissue [34, 35].

The cellular nature and differentiation stage of DFAT cells have not been fully clarified. However, several studies have suggested that DFAT cells are in the late stage of the differentiation process and classified them into preadipocytes [36, 37]. Similar to preadipocytes, DFAT cells could be redifferentiated into lipid-filled adipocytes under proper induction [29, 30, 38, 39]. Evaluations of stem cell-related markers and multilineage differentiation assays (i.e., adipogenesis, chondrogenesis, and osteogenesis) have also suggested that DFAT cells are similar to AdSCs [30, 38]. Poloni et al. also produced neurospheres from DFAT cells [40, 41], indicating that their multipotency might extend beyond the mesodermal lineages [40, 42]. Kishimoto et al. demonstrated that DFAT cells also showed proliferation and differentiation towards osteoblasts when they were cultured on self-assembling peptide or titanium fiber mesh scaffolds [14, 43, 44]. These findings highlight the hypothesis that DFAT cells are multipotent cells and have potential for use in tissue engineering.

**Figure 2:**
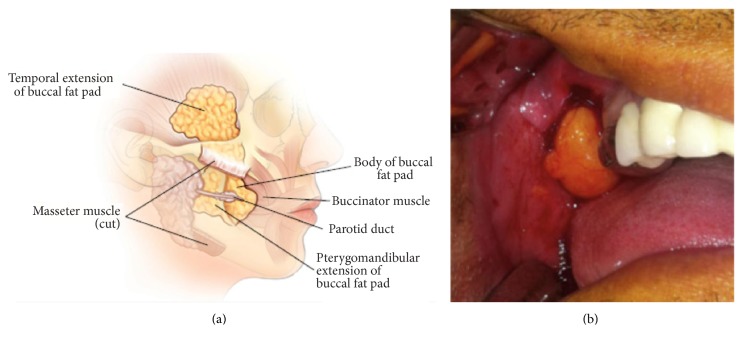
(a) Buccal fat pad anatomic location; permission granted from Muresan and Matarasso [92]. (b) Harvesting buccal fat pad with minimal discomfort for patients; permission granted from Khojasteh and Sadeghi [11].

One major goal of tissue engineering is to find a source of stem cells that can provide an adequate number for clinical application with minimal morbidity, maximal proliferation rate, and high differentiation potential [9, 17]. The BFP in the oral cavity is a mass of specialized fatty tissue that is distinct from subcutaneous fat and is located on either sides of the face between the buccinator muscle and other superficial muscles (Figure 2(a)) [45–48]. The BFP has a rich vascular supply [49–52] and can be harvested easily by an intraoral flap with minimal discomfort and complications for patients (Figure 2(b)) [9, 52–54]. In addition, BFP is a discarded tissue in plastic surgery for cheek reduction [13]. Furthermore, it is routinely administered in the treatment of the bone, periodontal defects [49, 55–59], congenital oroantral diseases, oronasal diseases [60], congenital cleft palate repair [61], oral submucous fibrosis [62, 63], intraoral malignant defects [64], and cheek mucosa defects [65].

Another advantage of BFP over subcutaneous fat is that its size appears to be similar among different people, independent of body weight and fat distribution [66]. Patients with little subcutaneous fat have BFP with normal weight and volume [9]. Recent studies have shown that both AdSCs [9, 10] and DFAT cells [14] isolated from human BFP (i.e., BFPSCs and BFP-DFAT cells) are similar to SC-AdSCs and possess high potential for regeneration of the bone and periodontal tissues [10, 12]. These properties may make the BFP a desirable cell source for tissue engineering and cell isolation.

### 4.1. Characteristics of BFPSCs

Since BFP is easily accessible via the oral cavity without injury to the external body surface, several research groups have recently evaluated the behavior of isolated AdSCs from the BFP as a proper source of adult cells for clinical applications [13].

#### 4.1.1. Morphology of Isolated BFPSCs

BFPSCs have been reported to remain in a quiescent state during 2–4 days of culture and showed spindle-shaped morphology similar to AdSCs, BMSCs, and USSCs [17]. Afterward, they began to multiply rapidly, formed a monolayer of large flat cells, and exhibited a more fibroblast-like morphology characteristic of AdSCs [9].

#### 4.1.2. BFPSC Surface Marker Profile

Cell surface markers on BFPSCs were characterized by immunofluorescence combined with flow cytometric analysis [10, 12, 67]. BFPSCs expressed MSC-defined markers, including CD73, CD90, and CD29, whereas they did not express lymphocyte or leucocyte antigens [12] and hematopoietic markers such as CD14, CD31, CD34 [9, 12], CD45 [10], CD19, and HLADR [9]. In addition, Traktuev et al. reported that BFPSCs showed some expression of CD34, which is characteristic of fresh AdSCs [68]. However, this marker declined with passage in AdSCs [9]. CD34^+^ cells have been shown to stimulate angiogenesis, and they are involved in neovascularization processes that facilitate healing of damaged tissues [69, 70].

Similar to other AdSCs, freshly isolated BFPSCs lack expression of CD105, but expression of this marker increases rapidly after seeding [20, 71]. AdSCs also usually lack expression of CD146, a characteristic marker of endothelial cells as well as vascular smooth muscle cells. However, Farré-Guasch et al. found a small population of CD146 cells in the first passages of BFPSCs [9]. The presence of this CD146-contaminated population, as well as the presence of CD34 cells, might be due to the highly enriched blood vessel supply in BFP [72]. This could be related to the excellent wound-healing properties of BFP as a pedicled graft in oral surgery for treatment of oroantral communications [55, 73], maxillary defects [51], oral submucous fibrosis [74], and vocal cord defects [75].

#### 4.1.3. Multilineage Differentiation Potential of BFPSCs

Several researchers have shown that BFPSCs are multipotent and differentiate towards osteogenic, adipogenic, and chondrogenic lineages in the presence of inductive stimuli [9, 12, 76, 77]. Broccaioli et al. showed that after 14 days of osteogenic induction, BFPSCs showed a significant upregulation of two specific markers, ALP activity and collagen deposition [12]. Farré-Guasch et al. also showed that after 1 week of culture in osteogenic medium, BFPSCs changed their morphology from spindle shaped to more polygon shaped, which was accompanied by an increase in ALP activity [9]. They reported that BFPSCs cultured in osteogenic medium showed osteocalcin expression. In addition, Niada et al. demonstrated the expression of core-binding factor alpha subunit 1 (CBFA1) and osteonectin. They also showed that compared with undifferentiated cells, osteogenic-differentiated BFPSCs derived from swine significantly increased the production of bone-specific markers, such as collagen, calcified extracellular matrix (ECM), ALP, and osteonectin [13].

After adipogenic induction of BFPSCs, the classic fibroblast-like shape of human AdSCs changed, and BFPSC populations showed intracellular lipid vacuoles that increased in size and number during culture [9, 12, 13]. Farré-Guasch et al. showed increased expression levels of a specific adipocyte marker during culture, peroxisome-proliferating receptor gamma (PPAR*γ*), reaching approximately four times higher induction compared to those of undifferentiated BFPSCs [9]. Some studies also showed that BFPSCs synthesized cartilage matrix molecules and produced an extracellular matrix characteristic for chondrocytes when grown in chondrogenic medium. Farré-Guasch et al. observed that after five days of chondrogenic induction, BFPSC morphology became more spheroid shaped [9]. Immunohistochemistry in differentiated BFPSCs indicated Toluidine blue-stained nodules indicative of a proteoglycan matrix characteristic for cartilage and expression of collagen II, a marker believed to be specific for articular cartilage. In addition, increased expression of the master chondrogenic factor, SOX9, was observed in BFPSCs, followed by decreased expression of the adipogenic marker PPAR*γ* when they induced towards the chondroblast lineage [9]. Niada et al. also observed GAG content in chondrogenic differentiated BFPSCs following three weeks of induction [13].

#### 4.1.4. Effect of Osteoinductive Agents on Bone Formation of BFPSCs

It is well known that rhBMP-2 enhances osteogenic differentiation of BMSCs [78]. However, it has been suggested that rhBMP-2 may not influence the osteogenic differentiation of AdSCs [25, 79]. Shiraishi et al. analyzed the capability of rhBMP-2 to induce osteogenesis on BFPSCs cultured in different culture conditions (i.e., osteoinductive reagents (OS), rhBMP2 (BMP), and the combination of BMP and OS (BMP-OS)) [10]. Their results indicated that rhBMP-2 strongly induced the osteogenic differentiation of human BFPSCs. After 10–14 days in culture, rhBMP-2 treatments (BMP and BMP-OS) induced distinct and substantial calcified-nodule formation and the expression of osteogenic markers in BFPSCs. In addition, they showed that BFPSCs pretreated with BMP-OS generated abundant bone tissue upon in vivo transplantation to the back subcutaneous pockets of nude mice [10].

Amelogenin (AM) is the most abundant enamel matrix protein, and it is favored for the repair of periodontal defects. Broccaioli et al. indicated that osteogenic differentiation of BFPSCs is specifically induced and upregulated by AM with a synergic effect with other osteoinductive factors [12], which was also reported for bone marrow MSCs [80–82]. This effect is more evident for BFPSCs than for SC-AdSCs, possibly due to the natural localization of BFPSCs, which could make them more prone to responding to stimuli naturally secreted in the same area of the body [12].

### 4.2. Comparison between BFPSCs and SC-AdSCs

Several research groups compared AdSCs derived from different areas of the body and evaluated their behavior in vitro to identify a convenient source for future preclinical studies (Table 1).

#### 4.2.1. Isolated Cell Yield

Several studies showed that the number of SC-AdSCs is similar to the number of BFPSCs, despite the different amounts of raw adipose tissues [12, 32]. However, Farré-Guasch et al. observed that the number of BFPSCs was two times higher than the number of SC-AdSCs after one week of culture. They admitted that this difference might be due to the differences in age, intrinsic characteristics of the patients, or particular properties of the adipose source [9].

#### 4.2.2. Size and Morphology

Several studies reported that both BFPSCs and SC-AdSCs showed similar morphology (fibroblast-like morphology) [9, 10, 13]. However, Broccaioli et al. observed that BFPSCs appeared slightly smaller and rounder than SC-AdSCs [12].

#### 4.2.3. Expression of Specific MSC Markers

Both types of cells similarly expressed defined MSC markers. Farré-Guasch et al. reported that the expression of CD34 was much higher in BFPSCs than in SC-AdSCs [9]. They also found the expression of CD146 in a small population of CD146 cells in the first passages of BFPSCs, but not in SC-AdSCs.

#### 4.2.4. Proliferation and Viability

Broccaioli et al. showed that SC-AdSCs proliferated faster than BFPSCs, with an average doubling time of 73 h compared to 126 h. This was also confirmed through a viability test, in which MTT incorporation by SC-AdSCs was mildly higher than that by BFPSCs [12]. However, similar doubling times were observed for BFPSCs and SC-AdSCs derived from swine [13].

#### 4.2.5. Colony Formation

Broccaioli et al. showed that BFPSCs were more prone to producing colonies than SC-AdSCs [12]. Interestingly, BFPSCs showed a significant increase in colony formation in late passages (from passages 7 to 9), suggesting a delayed selection of progenitor cells [12]. However, no significant difference has been observed between colony formation of porcine SC-AdSCs and BFPSCs from passages 1 to 4 [13].

#### 4.2.6. Differentiation

Several researchers showed that both cell types are multipotent and differentiate into different cell lineages in the presence of inductive stimuli [13, 76, 77]. Broccaioli et al. showed that after 14 days of osteogenic induction, both BFPSCs and SC-AdSCs showed a significant upregulation of two specific markers, ALP activity, and collagen deposition [12]. Niada et al. also showed that osteogenic-differentiated SC-AdSCs and BFPSCs derived from swine significantly increased the production of bone-specific markers compared with undifferentiated cells, such as collagen, calcified ECM, ALP, and osteonectin. The greatest difference was observed in the collagen level of 7-day-osteoinduced BFPSCs, which was 7 times higher than that of osteoinduced SC-AdSCs [13]. Similar capabilities in adipogenic and chondrogenic differentiation have been observed in both BFPSCs and SC-AdSCs [9, 13, 76].

#### 4.2.7. Effect of Human Serum on the Growth of BFPSCs and SC-AdSCs

Considering possible clinical applications, Broccaioli et al. studied the ability of BFPSCs and SC-AdSCs to grow in a medium supplemented with human serum [12]. Both populations were cultured in the presence of autologous serum (HAS) or heterologous serum (HHS), and their growth was compared to that of cells maintained in standard conditions (FBS). No differences in morphology were observed. In all the growth conditions, the AdSC populations maintained the fibroblast-like shape, and HAS induced a prompt increase in both BFPSC and SC-ASC numbers compared to other serums within 7 days. They noticed that the presence of human serum enhanced the proliferation rate of both cells types. This effect has not been previously observed in AdSCs, but similar heterogeneity in the response to autologous serum has also been described for BMSCs [26, 83–85] and could be explained by the differences in serum from the donors. Growing AdSCs in the presence of autologous serum could be a convenient and safe procedure in future cellular therapy, which would eliminate concerns about contact with animal proteins [12].

#### 4.2.8. BFPSC and SC-AdSC Culture and Osteogenic Differentiation on Biomaterials

Several research groups evaluated the behaviors of BFPSCs (e.g., adhesion, growth, and differentiation) on natural and synthetic biomaterials and compared them with other stem cells. Broccaioli et al. showed that both SC-AdSCs and BFPSCs can adhere to the autologous alveolar bone and periodontal ligament [12]. Moreover, they reported that these cells efficiently adhered to a collagen membrane. However, BFPSCs have not shown tight bonding to suture filaments of polyglycolic acid compared to AdSCs [12].

Niada et al. evaluated the ability of porcine AdSCs derived from SC and BFP to grow and differentiate on two synthetic scaffolds: titanium and plasma-treated silicon carbide [13]. They showed that both porcine AdSCs adhered and differentiated on these scaffolds. Ardeshirylajimi et al. assessed the osteogenic differentiation potential of MSCs on surface-modified poly(L-lactide) acid (PLLA) nanofibers. The MSCs were derived from four different sites: BFP, the bone marrow, subcutaneous adipose tissue, and unrestricted somatic stem cells [17]. No significant difference was observed in the proliferation rates. All four types of stem cells were demonstrated to differentiate efficiently into osteoblast-like cells on nanofibrous scaffolds in osteogenic medium. The highest ALP activity and calcium content were observed in BMSCs cultured on PLLA. Interestingly, BFPSCs resembled BMSCs in both ALP and calcium content. In addition, the highest expression of bone-related gene expression (i.e., Runx2, osteonectin, and osteocalcin) was observed in BFPSCs and BMSCs compared to that in other stem cell types [17].

### 4.3. Characteristics of BFP-DFAT Cells

AdSCs are used extensively for tissue engineering, and various studies have reported their utility [22–24]. However, AdSCs at passage 0 include contaminating endothelial cells, smooth muscle cells, and pericytes [86]. In contrast to AdSCs, mature adipocytes are the most abundant cell type in adipose tissue and have dynamic plasticity to be converted into DFAT cells [31]. Compared with ASCs, a relatively homogeneous cell population of DFAT cells has been revealed by flow cytometric analysis [31]. DFAT cells could not only redifferentiate into lipid-filled adipocytes in the same way as preadipocytes but they can also transdifferentiate into other cell types under proper conditions in vitro, including osteoblasts [14, 30, 38], chondrocytes [30], and myocytes [87–90]. In vivo studies also suggested that DFAT cells could regenerate fat pads, ectopic osteoid tissue, or muscle tissue [30, 36–38, 88, 90].

#### 4.3.1. Cell Surface Antigens

BFP-DFAT cells have been shown to be positive for CD90, CD105 [14], CD13, CD29, and CD44 [15] but negative for CD11b (monocyte marker), CD34 (hematopoietic progenitor cell marker), CD45 (leukocyte common antigen) [14], CD31, CD309, CD106, and alpha-smooth muscle actin [15, 30].

#### 4.3.2. The Osteogenic Differentiation

Kou et al. showed that after 3 weeks of osteogenic culture, the DFAT cells demonstrated limited mineralized matrix indicated by alizarin red S staining, while a relatively large portion of cells assumed a multilocular appearance and they were positive for adipose staining [15]. Previous reports confirmed the expression of osteogenic transcription factors in DFAT cells, including Runx2, osteopontin, osteorix, and osteocalcin [22, 29, 30]. However, Kou et al. showed that DFAT cells exhibited lower potential of differentiating towards osteoblasts than an adipocyte lineage [15]. This could be due to the fact that the multipotent capacity of DFAT cells is tissue specific [15].

#### 4.3.3. Small and Large DFAT Cells

Tsurumachi et al. divided adipocytes into two groups based on their size: those with cell diameters less than 40 *μ*m (small adipocytes: S-adipocytes) and those with diameters of 40–100 *μ*m (large adipocytes: L-adipocytes). They investigated the influence of the adipocyte size on the dedifferentiation efficiency into DFAT cells and compared the S- and L-DFAT cells based on the characteristics for MSCs. They showed that the S-adipocytes contained more juvenile adipocytes than the L-adipocytes. The results suggested higher rates of dedifferentiation for S-DFAT cells compared to those for L-DFAT cells and that the adipocyte size is positively associated with dedifferentiation. However, more studies are needed to reveal how the cell size could influence the efficiency of mature adipocyte dedifferentiation [16].

Tsurumachi et al. conducted flow cytometry and revealed higher CD146 expression in S-DFAT cells compared to that in L-DFAT cells, although both cells showed high expression levels of CD13, CD44, CD73, CD90, and CD105 [16]. S-DFAT cells showed higher osteogenic potential in particular compared to the L-DFAT cells. Similarly, a comparison between AdSCs and DFAT cells obtained from BFP demonstrated more effective induction of osteoblasts from DFAT cells than from AdSCs [14]. S-DFAT cells also exhibited higher osteogenic potential than L-DFAT cells. The results suggested that S-DFAT cells have an advantage over L-DFAT cells and AdSCs in bone tissue engineering.

### 4.4. Comparison between BFPSCs and BFP-DFAT Cells

Matsumoto et al. reported that human AdSCs at passage 1 are 13.3% positive for CD11b (monocyte marker) and 12.8% positive for CD45 (leukocyte common antigen). However, human DFAT cells at passage 1 are negative for these markers, indicating greater homogenicity of DFAT cells compared with AdSCs [30]. Kishimoto et al. showed that the expression of osteoblastic differentiation markers (BAP, OCN, and calcium) in BFP-DFAT cells was more prevalent than that in BFPSCs [14]. However, they indicated that the difference in the osteoblastic differentiation ability of BFPSCs and BFP-DFAT cells was not because of the difference of purity in the cell populations [14]. The same group also reported various osteoblastic differentiation abilities between human DFAT cells derived from the submandibular and human BMSCs [91]. Gene expression of Runx2, ALP, OCN expression, and calcium deposition was higher in DFAT cells than in bone marrow MSCs. More studies are needed to come to a general conclusion regarding the osteogenic capability of BFP-DFAT.

## 5. Conclusions

This study has reviewed the characteristics and osteogenic capability of AdSCs derived from BFP. This source of cells was also compared with other AdSCs from other parts of the body. BFP is an easily accessible source of stem cells that can be obtained easily via the oral cavity without injury to the external body surface. Its size is similar between people and independent of body weight and fat distribution. Comparing BFPSCs with other AdSCs showed similarities in cell yield, morphology, and multilineage differentiation. However, BFP has been shown to proliferate faster and is more prone to producing colonies. Limited studies have been conducted on the osteogenic capability of BFP-DFAT cells, which makes conclusions infeasible.

## Figures and Tables

**Table 1:** In vitro studies.

Authors	Study design	Cell source	Result
Farré-Guasch et al. (2010) [9]	Comparison:	Human	(i) Similar phenotype and morphology (spindle shaped)
	(i) BFPSCs		(ii) Able to differentiate into chondrogenic, adipogenic, and osteogenic lineages
	(ii) SC-AdSCs		
Broccaioli et al. (2013) [12]	Comparison:	Human	(i) Amelogenin: early osteoinductive factor for BFPSCs, but not SC-AdSCs
	(i) BFPSCs		(ii) Proliferation: both able to proliferate in presence of human serum and adhesion to scaffolds
	(ii) SC-AdSCs		(iii) Surface markers: both have a typical MSC immunophenotype
			(iv) Osteogenic and adipogenic differentiation: both show related markers (ALP activity, Coll deposition, and lipid vacuoles formation)
Niada et al. (2013) [13]	Comparison:	Swine	(i) No difference in proliferation, viability, and clonogenicity
	(i) BFPSCs		(ii) Differentiation: both have the ability to differentiate towards the osteoblast-like and adipocyte-like cells and also similar in size and granularity
	(ii) SC-AdSCs (cultured on titanium disks and silicon carbide-plasma)		(iii) Chondrogenic and osteogenic induction: both cells able to increase GAGs production over time and when osteoinduced on synthetic biomaterials, significantly increased amount of calcified ECM
			(iv) Seeded on titanium: increased amount of calcified ECM of about 46% and 37% for SC-AdSCs and BFPSCs, respectively
			(v) Seeded on silicon carbide: increased ECM deposition of 90% and 200% for SC-AdSCs and BFPSCs, respectively
Kishimoto et al. (2014) [14]	Comparison:	Human	(i) Surface markers: similar cell surface antigens of BFPSCs and BFP-DFAT cells
	(i) BFPSCs		(ii) Differentiation: osteoblastic differentiation ability of BFP-DFAT cells is higher than that of BFPSCs (OCN, Ca deposition, and alizarin red)
	(ii) BFP-DFAT cells		
Kou et al. (2014) [15]	Evaluation of BFP-DFAT cells	Human	(i) Differentiation: strong adipogenic but much weaker osteogenic capacity
			(ii) Surface markers: no expression of endothelial markers under angiogenic induction (NO VWF)
			(iii) Characteristics of BFP: similar to cells from abdominal subcutaneous adipose tissue
			(iv) Proliferation: no obvious decrease of proliferation or spontaneous differentiation up to the 25th passage
Tsurumachi et al. (2015) [16]	Evaluation of BFP-DFAT cells: Cells were dissociated by collagenase and centrifuged:	Human	(i) S cells: higher capacity to dedifferentiate into DFAT cells and more osteogenic differentiation ability
	(i) <40 *μ*m (S)		(ii) S- and L-DFAT cells had distinct characteristics
	(ii) 40–100 *μ*m (L)		(iii) High proportion of S-adipocytes in BFP
			(iv) S-adipocytes: more advantageous for inducing dedifferentiation into DFAT cells
Ardeshirylajimi et al. (2015) [17]	Comparison:	Human	(i) Proliferation: higher proliferation level in cells on PLLA-Bio but with no significant difference between stem cells
	(i) BFPSCs		(ii) BMSCs on PLLA-Bio: greatest ALP activity and mineralization (next close results: BFPSCs)
	(ii) BMSCs		(iii) Lowest ALP activity: AdSCs
	(iii) AdSCs		(iv) BFP: same osteogenic capacity as three other stem cells (S-spindle-shaped cells)
	(iv) USSCs		(v) Enzyme activities of BMSCs and BFPSCs: better on PLLA-Bio and PLLA
			(vi) Highest Ca deposition: PLLA-Bio
			(vii) Greater intracellular concentration: BMSCs
			(viii) Gene expression evaluation: highest expression of three bone-related genes: bioceramic-coated nanofibrous scaffolds

SC-AdSC: subcutaneous adipose stem cell; BFPSCs: buccal fat pad stem cells; MSCs: mesenchymal stem cells; ALP: alkaline phosphate; Coll.: collagen; GAG: glycosaminoglycan; ECM: extracellular matrix; DFAT: dedifferentiated fat; OCN: osteocalcin; Ca: calcium; VWF: von Willebrand factor; S: small; L: large; PLLA: poly L-lactic acid; BMSCs: bone marrow stem cells; USSCs: unrestricted somatic stem cells.

**Table 2:** In vivo studies.

Author	Study design	Cell source	Result
Shiraishi et al. (2012) [10]	An efficient method of generating bone from BFPSCs using rhBMP-2	Human	(i) BFPSCs can differentiate in vitro towards the osteoblastic lineage by addition of rhBMP-2 regardless of presence of osteoinductive reagents (ALP activity, calcification, and gene expression)
			(ii) Adipogenic genes were detectable only in cultures with rhBMP-2 and OSR.
			(iii) BFPSCs: formed engineered bone when pretreated with rhBMP-2 for inducing mature osteoblastic differentiation
			(iv) BFPSCs: had characteristic spindle shape and formed a monolayer
Nagasaki et al. (2015) [18]	Combination of LIPUS & NHA as scaffold for BFPSCs (transplantation in calvarial bone defects of nude mice)	Human	(i) Significantly increased the osteogenic differentiation of BFPSCs in vitro and in vivo
			(ii) Enhanced new bone formation of margin of defects
			(iii) Synergistic effects of LIPUS and NHA: capable of effectively inducing differentiation of BFPSCs into osteoblasts
Khojasteh and Sadeghi (2015) [11]	Preliminary: BFPSCs with autogenous iliac bone graft in treatment of maxillomandibular extreme jaw atrophy	Human	(i) Mean bone width change at the graft site: greater in the test group than in the control group (3.94–1.62 mm versus 3.01–0.89 mm)
			(ii) New bone formation: 65.32% in the test group versus 49.21% in the control group
			(iii) Increased amount of new bone formation & decreased secondary bone resorption in extensively atrophic jaws

BFPSCs: buccal fat pad stem cells; ALP: alkaline phosphate; NHA: nanohydroxyapatite; rhBMP2: recombinant human bone morphogenetic protein, LIPUS: low-intensity pulsed ultrasound; OSR: osteoinductive reagents.

**Table 3:** Comparison between BFPSCs and SC-AdSCs.

	Source	SC-AdSCs	BFPSCs	Ref
Volume of harvested adipose tissue	Human	37 ml	0.8 ml	[12]
Number of cells isolated form adipose tissue	Human	1.15 × 10^5^ (cells/ml)	1.1 × 10^5^ (cells/ml)	[12]
	Human	513 × 10^3^ (cells/gram) after 1 week of culture	253 × 10^3^ (cells/gram) after 1 week of culture	[9]
Number of cells	Human	1.3 × 10^6^ after 21 days, starting from 6 × 10^4^ AdSCs	5.9 × 10^5^ after 21 days, starting from 6 × 10^4^ AdSCs	[12]
Doubling time (h)	Human	73.5	126.5	[12]
	Swine	82.9	72.5	[13]
Morphology and size	Human	Fibroblast-like shape	Slightly smaller and rounder compared with SC-AdSCs	[12]
	Human	Fibroblast-like shape	Fibroblast-like shape	[13]
	Swine	Spindle-shaped morphology after seeding and fibroblast-like morphology after 7 days of culture	Similar to SC-AdSCs	[9]
Clonogenicity expressed as the percentage of cells able to produce CFU-F from passages 1 to 4	Human	—	9.2%	[12]
	Human	10.1%	8.9%	[13]
Expression of specific MSC markers	Human	CD73^+^, CD90^+^, and CD105^+^; CD14^−^, CD31^−^, and CD34^−^	Similar to SC-AdSCs	[12]
	Swine	(i) CD146^+^CD29^+^ was observed in initial passaged BFPSCs, but not SC-AdSCs		[9]
		(ii) CD34 was much higher in BFPSCs than in SC-AdSCs		
		(iii) CD105 was not observed in BFPSCs at first but increased by passaging the cells		
Collagen level increase by osteodifferentiated cells after 14 days of culture	Human	137.5%	74.5%	[12]
	Swine	87%	254%	[13]
ALP activity increase by osteodifferentiated cells after 14 days of culture	Human	553%	419%	[12]
	Swine	126%	201%	[13]
Adipogenic differentiation capacity after 14 days of culture	Human	No difference	No difference	[12]
GAGs content increase in chondrogenic-differentiated porcine cells after 21 days	Swine	184%	149%	[13]

CFU: colony-forming units; MSCs: mesenchymal stem cells; SC-AdSC: subcutaneous adipose stem cell; ALP: alkaline phosphate; GAG: glycosaminoglycan.
